# Fragment Libraries from Large and Novel Synthetic Compounds and Natural Products: A Comparative Chemoinformatic Analysis

**DOI:** 10.1021/acsomega.5c01420

**Published:** 2025-04-16

**Authors:** Verónica Ramírez-Cid, Ana L. Chávez-Hernández, Osvaldo Sánchez López, Raul Marques Novais, Temitayo Omowumi Alegbejo Price, Kamilla Moraes Alves, Wemenes J. Lima Silva, Flavio da Silva Emery, Carolina Horta Andrade, José L. Medina-Franco

**Affiliations:** † DIFACQUIM Research Group, Department of Pharmacy, School of Chemistry, Universidad Nacional Autónoma de México, Mexico City 04510, Mexico; ‡ Center for Research and Advancement in Fragments and Molecular Targets (CRAFT), School of Pharmaceutical Sciences at Ribeirao Preto, University of São Paulo, Ribeirão Preto, São Paulo 05508-060, Brazil; § Laboratory for Molecular Modeling and Drug Design (LabMol), Faculty of Pharmacy, Universidade Federal de Goiás, Goiânia Goiás 74605-170, Brazil; ∥ Center for Excellence in Artificial Intelligence (CEIA), Institute of Informatics, Universidade Federal de Goiás, Goiânia Goiás 74605-170, Brazil

## Abstract

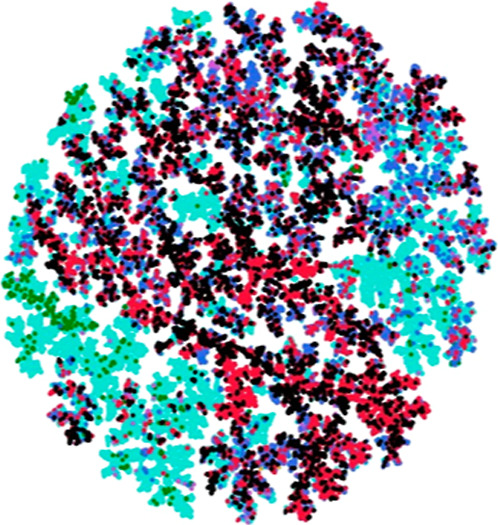

We report comprehensive fragment libraries obtained from large natural product databases and compare their chemical space coverage and diversity with those of synthetic fragment libraries. Specifically, we obtained 2,583,127 fragments derived from the recently updated collection of open natural product (COCONUT) data set with more than 695,133 unique (nonduplicate) natural products and 74,193 fragments derived from the Latin America Natural Product Database (LANaPDB) with 13,578 unique natural products from Latin America. The content, chemical space coverage, and chemical diversity of the natural product libraries were compared to the recently developed CRAFT library, which contains 1214 fragments based on distinct heterocyclic scaffolds and natural product-derived chemicals. The fragment libraries herein obtained and curated are freely available at https://github.com/DIFACQUIM/Fragment-libraries-from-large-synthetic-compounds-and-natural-products-collections.git.

## 1 Introduction

Major sources of small molecule drugs are natural products (NPs) and chemical synthesis (mostly organic synthesis). Historically, humans have used NP, particularly plants, in the treatment of different diseases.^1−3^ An overall challenge developing NP-based drugs is their structural complexity that is frequently associated with the difficulty to synthesize them. Continued efforts have been made to design compounds that resemble NP and are rich in sp^3^ atoms.^4−6^ An approach is to deconstruct NP into fragments and combine unrelated NP fragments into pseudo-NP using synthetic methods.^5,7,8^

Over the past few years, efforts have been made to compile a list of the NPs. For instance, the second version of the COlleCtion of Open Natural prodUcTs (COCONUT) has 695,133 distinct structures.^9,10^ Similarity, Latin American countries have been assembling a unified and open-access Latin American Natural Product Database (LANaPDB) that gathers ten databases.^11^ The first version of LANaPDB had 12,959 chemical structures^12^ and the most recent version has 13,578 compounds.^13^

The integration of fragment-based drug design (FBDD) with other drug discovery approaches has become crucial in the development of new candidates. The increasing application of biophysical and biochemical techniques for fragment identification, alongside rational library design, facilitates the transformation of fragments into promising chemical series. The significance of FBDD underscores its vital role in fostering innovative therapies and broadening the horizons of medicinal chemistry.^14,15^

FBDD typically utilizes small organic molecules with fewer than 20 non-hydrogen atoms, adhering to the “rule of three” (RO3), ensuring a more efficient exploration of chemical space.^14,16^ Sources such as NPs and synthetic libraries^17^ further enhance this process by providing fragments with ideal physicochemical properties for the creation of lead series.

Unlike HTS, which screens hundreds of thousands of molecules, FBDD relies on smaller libraries, typically comprising 1000 to 5000 low-molecular-mass fragments. This enables more efficient screening, reduced operational costs, and greater practicality in library management. Over the past two decades, FBDD has achieved remarkable milestones, including the approval of drugs (for example: venetoclax,^18^ vemurafenib,^19^ sotorasib,^17^ and pexidartinib^20^) and the development of over 40 compounds in clinical stages originating from FBDD screenings. This success underscores its significance in addressing various diseases.^21,22^

For computer-guided FBDD, fragments can be obtained through deconstruction methods such as REtrosynthetic Combinatorial Analysis Procedure (RECAP),^23^ Breaking of Retrosynthetically Interesting Chemical Substructures (BRICSs),^24^ and MOlecule fRagmenTAtion fRamework (MORTAR),^25^ a framework integrated with three algorithms: ErtlFunctional GroupsFinder,^26^ Sugar Removal Utility, and Scaffold Generator.^27^ Fragmentation algorithms identify characteristic molecular structures in a comprehensible way based on structural definitions like functional groups; therefore, the main advantage of fragmentation algorithm RECAP regarding MORTAR algorithms (scaffold generations or group finder) is that a fragment captures information both molecular scaffolds and functional groups.^25^

Another method of obtaining molecular fragments for computer- and experimentally guided FBDD is from commercial vendors or academic groups. Commercial vendors have collections of this type of building blocks, for example, Enamine (12,000 fragments),^28^ ChemDiv (74,000 fragments),^29^ Maybridge (30,000 fragments),^30^ and Life Chemicals (65,000 fragments).^31^

The center for research and advancements in fragments and molecular targets (CRAFT) has made available its synthetic database of compounds and fragments at https://github.com/CRAFT-Therapeutics/Fragment-library.git. CRAFT is a Brazilian interinstitutional collaboration between the University of Sao Paulo and the Federal University of Goias. A major goal of CRAFT is to advance drug discovery, with a special emphasis on neglected infectious diseases as well as emerging diseases such as bacterial and viral infections.^32^ CRAFT has compiled a fragment library containing structures based on new heterocyclic scaffolds and compounds derived from NPs.^33^ All the fragments in the CRAFT library were obtained experimentally, and this plays a main role in lead discovery^34^ because some computer-designed compounds cannot be synthesized.^35^ Molecules generated from the CRAFT’s fragments are designed to be synthetically accessible.^32^

The goal of this study was to analyze the contents, properties, and chemical diversity of fragment libraries obtained from two large natural product libraries (COCONUT 2.0 and the most updated version of LANaPDB), chemical synthesis (CRAFT), and commercial fragment libraries (Enamine, ChemDiv, Maybridge, and Life Chemicals).

## 2 Methodology

### 2.1 Data Sets

Table 1 summarizes the data sets used in this work. COCONUT is a large library with the chemical structures and annotations of 695,133 NPs and is a compendium of other publicly available NP collections including LANaPDB. The latest update of LANaPDB has 13,578 nonduplicate NPs. Table 2 summarizes the number of compounds in CRAFT and the commercial and synthetic fragment libraries. CRAFT’s fragments contain 1214 fragments obtained by chemical synthesis. Commercial fragment libraries were 12,505 Enamine’s fragments with solubility in water, 74,721 ChemDiv’s fragments, 30,099 Maybridge’s fragments, and 65,552 Life Chemical’s fragments.

**Table 1:** Natural Product Libraries Studied in This Work

data set	initial size	size after standardization protocol	initial number of fragments^a^	fragments after standardization protocol	fragments that fulfill the RO3 (percentage)	refs
LANaPDB	13,578	13,578	74,193	74,193	1832 (2.5)	(13,36)
COCONUT	695,133	648,721	2,583,127	2,583,127	38,747 (1.5)	(9,10)

a Compounds with molecular weight larger than 1000 Da were excluded.

**Table 2:** CRAFT Fragment Library and Commercial Fragment Libraries from Chemical Vendors

data set	initial fragments	number of fragments after standardization protocol	fragments that fulfill all properties of the RO3 (percentage)	refs
CRAFT	1214	1202	176 (14.6)	(32)
Enamine (soluble in water)	12,505	12,496	8386 (67.1)	(28)
ChemDiv	74,721	72,356	16,723 (23.1)	(29)
Maybridge	30,099	29,852	5912 (19.8)	(30)
Life Chemicals	65,552	65,248	14,734 (22.6)	(31)

### 2.2 Data Set Standardization

Fragment libraries were stored using simplified Molecular Input Line Entry System (SMILES) strings.^37^ The fragments were prepared and curated using toolkits RDKIT version (2024.03.5)^38^ and MolVS version 0.1.1.,^39^ and standardization protocol was described by Sánchez-Cruz et al.^40^ Fragments were selected if they had the elements H, B, C, N, O, F, Si, P, S, Cl, Se, Br, and I. Fragments with multiple components were split, retaining the largest component. Then, fragments were reionized and neutralized, and a canonical tautomer was generated. Finally, unique fragments were retained.

### 2.3 Fragment Libraries

NP fragment libraries were generated for the compounds in COCONUT^10^ and LANaPDB^13^ with molecular weight less than 1000 Da. This threshold was selected to guarantee the fragmentation of at least 95% of the compounds, also because fragmentation of larger molecules takes significantly longer. The fragments were obtained using the RECAP^23^ function from the RDKit toolkit. RECAP breaks eleven chemical bonds as amine, amide, ester, urea, olefin, ether, aromatic nitrogen–aliphatic carbon, lactam nitrogen–aliphatic carbon, aromatic carbon–aromatic carbon, quaternary nitrogen, and sulfonamide. The synthetic fragment library was obtained from CRAFT (available at https://github.com/CRAFT-Therapeutics/Fragment-library.git).^32^ We also gathered four commercial fragment libraries, summarized in Table 1.

Fragments fulfilling the RO3^16^ were retained and are referred to in this manuscript as “Fragment RO3”. The RO3 rule describes fragments that have six properties of pharmaceutical relevance: molecular weight (MW), rotatable bonds (RBs), topological polar surface area (TPSA), partition coefficient octanol/water (Log P), hydrogen-bond acceptors (HBAs), and hydrogen-bond donors (HBDs), with the values: MW ≤ 300 Da, RB ≤ 3, TPSA ≤60 Å^2^, logP ≤3, HBA ≤3, and HBD ≤3.

### 2.4 Synthetic Accessibility Score

The synthetic accessibility score (SA score) is a calculated value that approximates the feasibility of a molecule to be synthesized.^34^ SA score is calculated by the difference between fragment score and complexity penalty. The fragment score indicates the structural feature viability of synthesized molecules and is calculated as a sum of contributions of all fragments contained in the molecule. The complexity score is the sum of ring complexity (ring bridge atoms and spiro atoms), large rings, stereocenters, and molecular size.^41,42^ The SA score was computed using the Python script of Ertl and Schuffenhauer.^34^

### 2.5 Content, Complexity, and Structural Diversity

Fragments and “Fragment RO3” were analyzed using fourteen constitutional and complexity descriptors (cf. Table 6). Their structural diversity was measured using the Tanimoto coefficient^43^ and the following fingerprints: Molecular ACCes System (MACCS) keys (166 bit)^44^ and Morgan fingerprints^45^ with radius 2 (Morgan2, 1024 bit) and radius 3 (Morgan3, 1024 bit).

**Table 3:** Unique and Overlapping Fragments between CRAFT and the Reference Libraries

data set	COCONUT	LANaPDB	Enamine	ChemDiv	Maybridge	Life Chemicals
unique fragments present in the CRAFT’s fragment library^a^	1139 (94.76%)	1199 (99.75%)	1195 (99.42%)	1152 (95.84%)	1179 (98.09)	1178 (98.00%)
overlapping structures with CRAFT’s fragments	63 (5.24%)	3 (0.25%)	7 (0.58%)	50 (4.16%)	23 (1.91%)	24 (2.00%)
unique fragments present in the CRAFT’s “Fragment RO3”^b^	144 (81.82%)	175 (99.43%)	171 (97.16%)	153 (86.93%)	163 (92.61%)	168 (95.45%)
overlapping structures with CRAFT’s “Fragment RO3”	32 (18.18%)	1 (0.57%)	5 (2.84%)	23 (13.07%)	13 (7.39%)	8 (4.55%)

a 1202 CRAFT’s fragments.

b 176 CRAFT’s “Fragment RO3”.

**Table 4:** Unique and Overlapping Fragments between LANaPDB and the Reference Libraries

data set	COCONUT	CRAFT	Enamine	ChemDiv	Maybridge	Life Chemicals
unique fragments present in the LANaPDB’s fragments^a^	38,815 (52.32%)	74,190 (99.996%)	74,167 (99.96%)	74,147 (99.94%)	74,149 (99.94%)	74,172 (99.97%)
overlapping fragments with LANaPDB’s fragments	35,378 (47.68%)	3 (0.004%)	26 (0.04%)	46 (0.06%)	44 (0.06%)	21 (0.03%)
unique fragments present in the LANaPDB’s “Fragment RO3”^b^	459 (25.05%)	1831 (99.95%)	1809 (98.74%)	1803 (98.42%)	1802 (98.36%)	1815 (99.07%)
overlapping fragments with LANaPDB’s “Fragment RO3”	1373 (74.95%)	1 (0.05%)	23 (1.26%)	29 (1.58%)	30 (1.64%)	17 (0.93%)

a 74,193 LANaPDB’s fragments.

b 1832 LANaPDB’s “Fragment RO3”.

**Table 5:** Unique and Overlapping Fragments between COCONUT and the Reference Libraries

data set	CRAFT	LANaPDB	Enamine	ChemDiv	Maybridge	Life Chemicals
unique fragments present in the COCONUT’s fragments^a^	2,583,064 (99.997%)	2,547,749 (98.63%)	2,582,782 (99.99%)	2,580,338 (99.89%)	2,581,975 (99.96%)	2,582,240 (99.97%)
overlapping fragments with COCONUT	63 (0.003%)	35,378 (1.37%)	345 (0.01%)	2789 (0.11%)	1152 (0.04%)	887 (0.03%)
unique fragments present in the COCONUT “Fragment RO3”^b^	38,715 (99.92%)	37,374 (96.46%)	38,497 (99.35%)	37,171 (95.93%)	38,014 (98.11)	38,231 (98.67%)
overlapping fragments with COCONUT RO3	32 (0.08%)	1373 (3.54%)	250 (0.65)	1576 (4.07%)	733 (1.89%)	516 (1.33%)

a 2,583,127 COCONUT’s fragments.

b 38,747 COCONUT’s “Fragment RO3”.

### 2.6 Chemical Space and Chemical Multiverse Analysis

Chemical space can be defined as an *M*-dimensional Cartesian space, each of the dimensions encoding a molecular descriptor of a set of molecules.^46^ The number of descriptors describes the number of dimensions that make up this chemical space, and the type or nature of the descriptors defines the specific type of chemical space (e.g., property-based and fingerprint-based chemical space). The chemical multiverse concept is a natural extension of the chemical space and it can be defined as a group of alternative chemical spaces of a set of molecules, each defined by a different set of molecular descriptors.^47^ In this study, the chemical space visualization was done using the fingerprints Morgan2 (1024 bit), Morgan3 (1024 bit), and MACCS keys (166 bit) and two algorithms: Tree MAP (TMAP)^48^ and T-distributed Stochastic Neighbor (t-SNE).^49^ TMAP is grouped hierarchically as compounds according to its common structures using a molecular fingerprint, nearest-neighbor algorithm (*k*), and query algorithm (*k*_c_). TMAP was generated using *k* = 50 and *k*_c_ = 10. t-SNE was generated using the number of nearest neighbors (perplexity = 40) and the number of iterations (n_iter = 300).

## 3 Results and Discussion

Table 1 summarizes the number of compounds before and after the standardization protocol (Section 2.2) and the number of fragments generated for the two NP libraries. It is noteworthy that there is a large number of fragments generated from COCONUT (more than 2.5 million). For both NP libraries, the percentage of “Fragment RO3” is relatively small (2.5% and 1.5% for LANPDB and COCONUT, respectively). Table 2 summarizes the number of total fragments and “Fragment RO3” for CRAFT and the four fragment libraries of chemical vendors. Enamine, ChemDiv, and Life Chemicals had a higher percentage of “Fragment RO3” (67.1%, 23.1%, and 22.6%, respectively) than Maybridge and CRAFT (19.8% and 14.6%, respectively). Not surprisingly, the proportion of fragments that fulfill the RO3 in the chemical vendors (between 22.6% and 67.1%) is higher than the same type of fragments in NP libraries (1.5% and 2.5%).

### 3.1 Unique Natural Product Fragments

**Figure 1:**
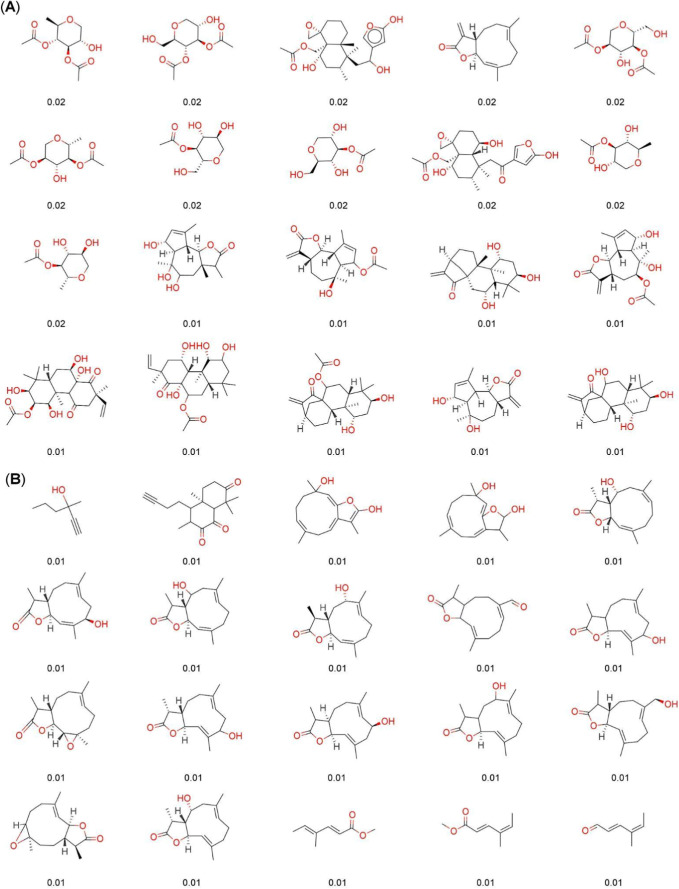
Chemical structures were done with Marvin 17.21.0^50^ of the twenty most frequent and unique (A) LANaPDB’s fragments and (B) LANaPDB’s “Fragment RO3”. The percentage of each fragment is indicated below each structure.

**Figure 2:**
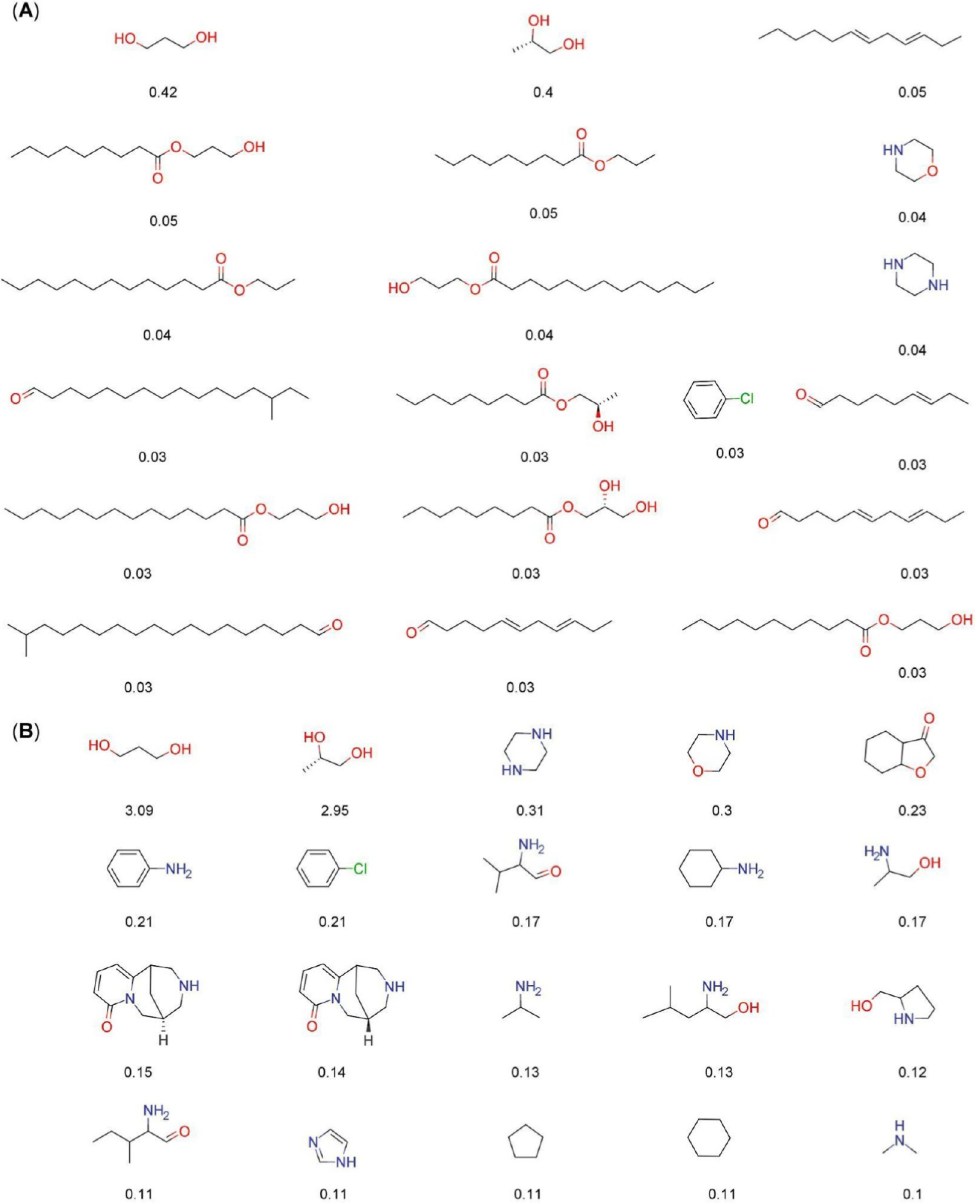
Chemical structures of the twenty most frequent and unique (A) COCONUT’s fragments and (B) COCONUT’s “Fragment RO3”. The percentage of each fragment is indicated below each structure.

Figures 1 and 2 show the ten most frequent chemical structures from LANaPDB’s fragments, LANaPDB’s “Fragment RO3” (Figure 1), and COCONUT’s fragments and COCONUT’s “Fragment RO3” (Figure 2). The percentage of each fragment is indicated below for each chemical structure. LANaPDB’s fragments (Figure 1A) had the most fused bicycles (twelve different and unique fragments with around 0.02% and 0.01%) and bridged bicycles compared with COCONUT’s fragments.

Fragments fulfilling RO3 showed noticeable differences. For example, LANaPDB’s “Fragment RO3” (Figure 1B) had the most fused bicycles and macrocycles (sixteen different fragments with 0.01% each one and around ten atoms). LANaPDB’s fragments had more bridged bicycles (three different and unique fragments with around 0.01%) than LANaPDB’s “Fragment RO3”.

The most frequent fragments from COCONUT (Figure 2A) are aliphatic compounds, mostly esters (nine fragments with 0.04%–0.05%) and ketones (five fragments with 0.03%). In contrast, the chemical structures of the twenty most frequent “Fragment RO3” obtained from COCONUT (Figure 2B) had a larger diversity and contained aliphatic alcohols (3.09–2.95%), aromatic rings (0.21%), bridge bicycles (0.14%–0.15%), and aliphatic cycles (0.17%–0.11%). It is noteworthy the high number of oxygen-containing fragments and the presence of very small fragments or mini fragments.

### 3.2 Common Fragments between Natural Product Libraries

**Figure 3:**
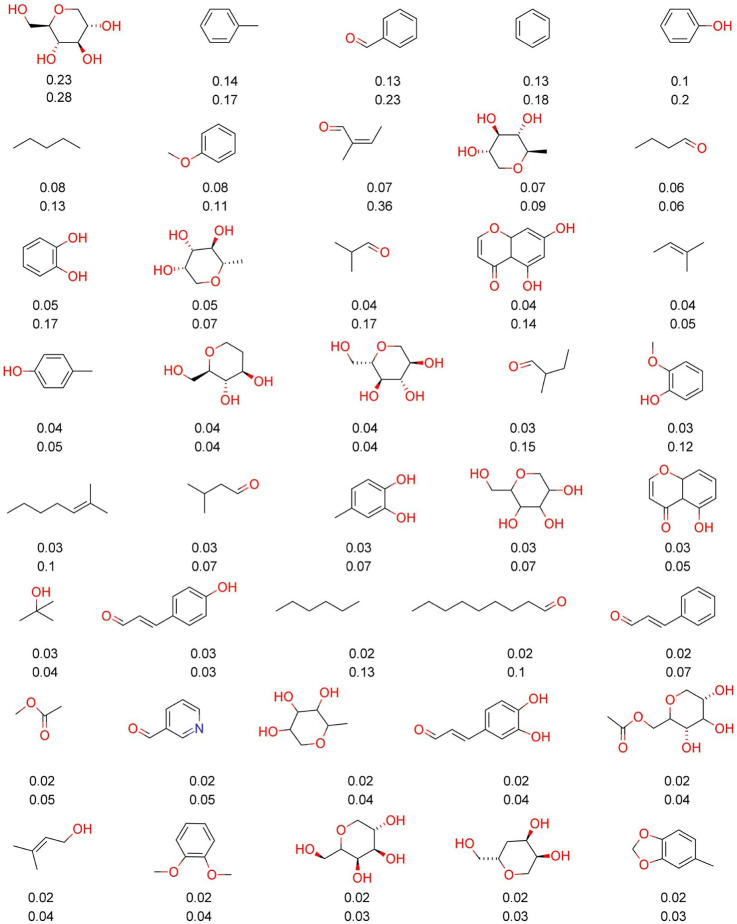
Forty most frequent and common fragments between the fragment libraries of LANaPDB and COCONUT generated in this study. Fragments with MW between 70 and 300 Da were considered. The percentage of each fragment is indicated below each chemical structure (proportion in COCONUT is the number on top). Marvin 17.21.0 was used for drawing the chemical structures.^50^

**Figure 4:**
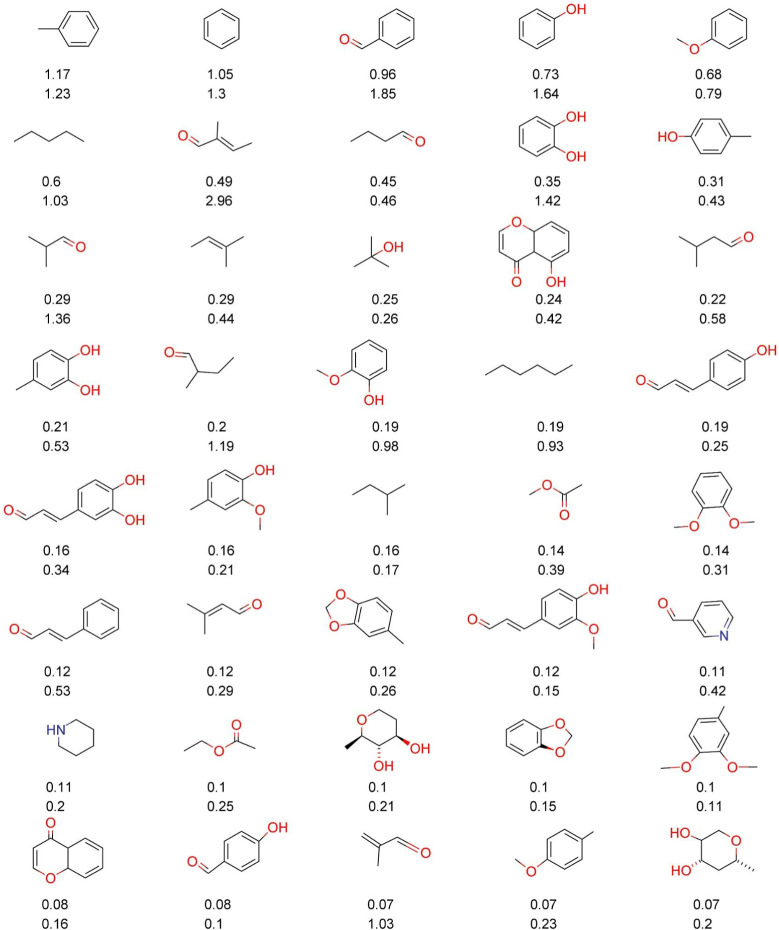
Forty most frequent and common fragments between the “Fragment RO3” libraries of LANaPDB and COCONUT generated in this study. Fragments with MW between 70 and 300 Da were considered. The percentage of each fragment is indicated below each chemical structure (proportion in COCONUT is the number on top). Marvin 17.21.0 was used for drawing the chemical structures.^50^

Figure 3 and 4 show the forty most frequent fragments and “Fragment RO3” between COCONUT and LANaPDB. Figures 3 and 4 consider fragments with MW between 70 and 300 Da. The percentage of each fragment is indicated below each chemical structure. The first value (top) is for COCONUT and the second (bottom) is for LANaPDB. Figure 3 shows that the common structures between COCONUT and LANaPDB’s fragments were tetrahydropirans (0.23% and 0.28%), toluene (0.14% and 0.17%), benzene (0.13% and 0.18%), phenol (0.1% and 0.2%), anisol (0.08% and 0.11%), benzopyrans (0.04% and 0.14%), and nicotinaldehyde (0.02% and 0.05%). Figure 4 indicates that the common “Fragments RO3” between COCONUT and LANaPDB (Figure 4) were tetrahydropirans (0.24% and 0.42%), ophiocerin B (0.07% and 0.02%), nicotinaldehyde (0.11% and 0.42%), piperidine (0.11% and 0.2%), 1,3-benzodioxole (0.1% and 0.15%), and derivatives of 3-methoxyphenyl (0.07% and 0.23%).

Tables 3–5 summarize the number of unique and common fragments and “Fragment RO3” for the CRAFT, LANaPDB, and COCONUT libraries, as compared to the reference fragment collections.

Table 3 indicates that CRAFT has a high percentage of unique fragments (94.76%–99.75%) and “Fragment RO3” (81.82%–99.43%) as compared to COCONUT, LANaPDB, ChemDiv, Enamine, Maybridge, and Life Chemicals. COCONUT (63, 5.24%) was the database with the highest percentage of fragments in common with CRAFT. Similarly, CRAFT and COCONUT have 32 (18.18%) “Fragment RO3” in common. In contrast, LANaPDB shared the fewest structures with CRAFT. CRAFT has a high percentage of 99.75% (1199) fragments and 99.43% (175) “Fragment RO3” not present in LANaPDB. Only 1 “Fragment RO3” is common between LANaPDB and CRAFT.

Table 4 shows that LANaPDB has a high number of fragments (47.68%) and “Fragment RO3” (74.95%) in common with COCONUT, which is to be expected since COCONUT integrates the first version of LANaPDB. With respect to the other reference libraries, LANaPDB maintains more than 99.9% of its fragments and more than 98% of “Fragment RO3” unique.

Table 5 reports the number of unique and overlapping fragments between COCONUT and the reference libraries. The percentage of unique fragments is in the range of 98.63%–99.99% and “Fragment RO3” is between 95.93% and 99.92%. COCONUT had the highest number of fragments in common with LANaPDB (35,378). However, ChemDiv has 1576 (4.07%) “Fragment RO3” in common with COCONUT, even more than those between LANaPDB and COCONUT (1373 “Fragment RO3”, 3.54%).

On the other hand, the fraction of NP “Fragment RO3”, LANaPDB RO3, and COCONUT RO3, present in fragment libraries commercially available from vendors (Enamine, ChemDiv, Maybridge, and Life Chemicals), was low. For instance, LANaPDB “Fragment RO3” (0.93%–1.64%) and COCONUT “Fragment RO3” (0.65%–4.07%) were low regarding to CRAFT’s “Fragment RO3” (2.84%–13.07%). The fragments obtained experimentally by both institutes or commercial vendors can support the lead discovery.^34^

**Figure 5:**
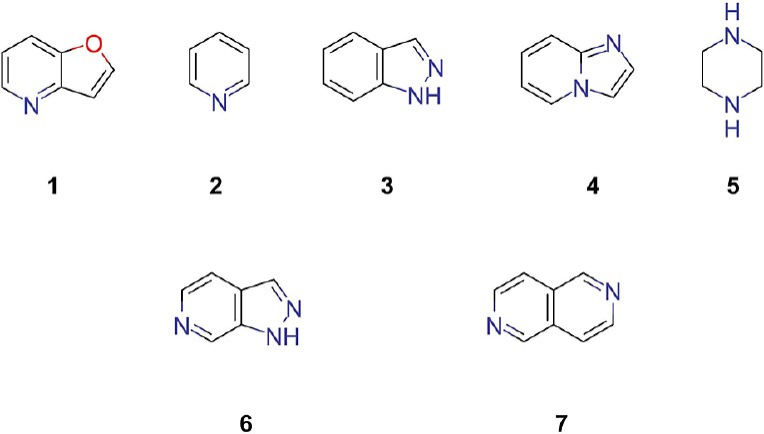
Top five most common scaffolds in the CRAFT fragment library and examples of some novel scaffolds contained in this library.

The number of unique fragments indicates a higher degree of structural diversity. The NP databases are diverse, with respect to the reference databases. Similarly, CRAFT shows diversity, although it shares more fragments and “Fragment RO3” in common with COCONUT than with other libraries. The reason for this could be that overlapping the fragments that comprise CRAFT are NP derived.^32^ This diversity is clearly reflected in CRAFTs’ library, which incorporates a wide array of structural frameworks derived not only from natural and semisynthetic compounds but also synthetic products. The library includes compounds featuring fused-ring systems, encompassing a broad spectrum of structures that spans from commonly found ring systems (e.g., pyridine) to innovative bicycles (e.g., pyrazolo[1,5-*c*]pyrimidine). Such a varied collection of scaffolds underscores the library’s potential for occupying a wide range of the available chemical space. More specifically, the CRAFT fragment library revealed a total of 122 distinct scaffolds, highlighting the rich structural diversity within the library. Among these, the top five scaffolds after benzene are furo[3,2-*b*]pyridine 1, pyridine 2, indazole 3, imidazo[1,2-*a*]pyridine 4, and piperazine 5 (Figure 5).

These scaffolds represent important structural motifs commonly encountered in bioactive molecules.^51^ Notably, other significant scaffolds included 1*H*-pyrazolo[3,4-*c*]pyridine 6 and 2,6-naphthyridine 7 (Figure 5), which exemplifies novel scaffolds synthesized previously by our group.^52^ Further analysis indicated that only 10 of the 122 scaffolds were nonheterocyclic, while 88 contained nitrogen within their core structure, suggesting a strong emphasis on heterocyclic chemistry. Moreover, 77 of these scaffolds were fused, further supporting the hypothesis that many of them closely resemble natural product structures, which are often characterized by fused-ring systems. This unique composition not only broadens the chemical diversity of the library but also explains why it demonstrates a higher overlap with the COCONUT database compared with other chemical libraries, providing a richer source for exploring bioactive and functional molecular scaffolds.

It is useful to identify the relation between the different databases for the identification of structures conserved in the different databases. However, the diversity in the libraries makes it possible to generate a database of the collections and opens the possibility of their analysis in terms of various chemical and biological implications.^53^

### 3.3 Structural Content, Composition, and Complexity of the Compound and Fragment Libraries

As described in the Methodology section, fragments and “Fragment RO3” were analyzed using fourteen constitutional and complexity descriptors. Table 6 describes the constitutional and complexity descriptors of NPs from the LANaPDB and the COCONUT. Tables 7 and 8 summarize constitutional and complexity descriptors from fragments and “Fragment RO3”. The fraction of carbon, oxygen, and nitrogen atoms for LANaPDB’s compounds was 0.8, 0.19, and 0.01, respectively, and close to COCONUT’s compounds 0.78, 0.18, and 0.04, respectively. Chavez-Hernandez et al. (2024) reported the structural composition of NPDBEjeCol-, BIOFACQUIM-, NuBBEDB-, PeruNPDB-, and FDA-approved drugs. Similar to this study’s COCONUT and LANaPDB, all databases of NPs maintain a tendency for a higher fraction of carbon atoms, presenting a higher content of oxygen atoms than nitrogen atoms. While this behavior is distinct in libraries that contain synthetic compounds, for example, as FDA-approved drugs (the fraction of carbon 0.68, oxygen atoms 0.17, and nitrogen 0.09).^54^ This corresponds to what has been reported in the literature.^55,56^

**Table 6:** Constitutional and Complexity Descriptors of NPs^a^

data set	COCONUT	LANaPDB
carbon atoms	28.27	21.34
oxygen atoms	6.6	5.43
nitrogen atoms	1.26	0.21
fraction of carbons	0.78	0.8
fraction of oxygens	0.18	0.19
fraction of nitrogens	0.04	0.01
fraction of sp^3^ carbons	0.57	0.59
fraction of chiral carbons	0.18	0.21
molecular weight	510.91	374.69
heavy atoms	36.35	27
rings	3.74	3.62
aliphatic rings	2.35	2.64
aromatic rings	1.39	0.98
heterocycles	1.62	1.27
aliphatic heterocycles	1.19	1
aromatic heterocycles	1.39	0.98
spiro atoms	0.18	0.35
bridgehead atoms	0.47	0.55

a Mean value of the distribution.

**Table 7:** Constitutional and Complexity Descriptors of NP Fragments and Reference Libraries^a^

data set	COCONUT	LANaPDB	CRAFT	Enamine	ChemDiv	Maybridge	Life Chemicals
carbon atoms	25.95	24.48	14.90	10.55	12.20	12.77	12.59
oxygen atoms	10.11	8.11	2.22	1.41	2.05	1.86	1.76
nitrogen atoms	0.41	0.22	2.38	2.02	2.20	2.11	2.86
fraction of carbons	0.71	0.75	0.72	0.70	0.70	0.70	0.70
fraction of oxygens	0.27	0.24	0.11	0.10	0.12	0.10	0.10
fraction of nitrogens	0.01	0.01	0.12	0.14	0.13	0.12	0.16
fraction of sp^3^ carbons	0.64	0.71	0.18	0.42	0.31	0.25	0.41
fraction of chiral carbons	0.29	0.32	0.01	0.04	0.02	0.01	0.03
molecular weight	517.31	460.51	291.65	215.12	249.30	264.73	252.55
heavy atoms	36.55	32.82	20.54	15.07	17.20	18.00	17.86
rings	3.38	3.96	2.79	1.87	2.10	2.15	2.37
aliphatic rings	2.36	3.31	0.61	0.70	0.41	0.36	0.79
aromatic rings	1.01	0.65	2.18	1.17	1.69	1.79	1.58
heterocycles	1.73	1.45	1.50	1.14	1.12	0.99	1.69
aliphatic heterocycles	1.5	1.18	0.51	0.53	0.32	0.24	0.59
aromatic heterocycles	1.01	0.65	2.18	1.17	1.69	1.79	1.58
spiro atoms	0.15	0.75	0.01	0.01	0.01	0.01	0.01
bridgehead atoms	0.51	1.52	0.02	0.02	0.03	0.05	0.06

a Mean value of the distribution.

**Table 8:** Constitutional and Complexity Descriptors of NPs and Reference Libraries Following RO3^a^

data set	COCONUT	LANaPDB	CRAFT	Enamine	ChemDiv	Maybridge	Life Chemicals
carbon atoms	9.97	10.04	9.7	10.61	9.44	9.52	11.04
oxygen atoms	1.82	2.19	1.15	1.25	1.16	1.14	1.22
nitrogen atoms	0.64	0.14	1.76	1.77	1.6	1.49	1.9
fraction of carbons	0.78	0.8	0.72	0.72	0.73	0.72	0.73
fraction of oxygens	0.15	0.18	0.09	0.09	0.09	0.09	0.08
fraction of nitrogens	0.06	0.01	0.14	0.12	0.13	0.12	0.13
fraction of sp^3^ carbons	0.55	0.6	0.16	0.44	0.38	0.28	0.47
fraction of chiral carbons	0.14	0.17	0.01	0.05	0.03	0.02	0.04
molecular weight	177.67	173.01	195.81	210.09	189.9	192.86	213.67
heavy atoms	12.57	12.39	13.34	14.76	12.88	13.04	14.99
rings	1.54	1.51	1.88	1.84	1.61	1.66	2.06
aliphatic rings	1	1.18	0.38	0.78	0.49	0.44	0.95
aromatic rings	0.54	0.33	1.51	1.07	1.12	1.23	1.1
heterocycles	0.73	0.61	1.1	1.06	0.88	0.79	1.32
aliphatic heterocycles	0.54	0.54	0.3	0.59	0.36	0.27	0.69
aromatic heterocycles	0.54	0.33	1.51	1.07	1.12	1.23	1.1
piro atoms	0.05	0.07	0	0.02	0.01	0.01	0.02
bridgehead atoms	0.17	0.14	0	0.02	0.04	0.08	0.11

a Mean value of the distribution.

The fraction of carbon, nitrogen, and oxygen atoms for LANaPDB’s fragments was 0.75, 0.24, and 0.01, respectively, and was similar to that of LANaPDB’s “Fragment RO3” (0.8, 0.18, and 0.01, respectively). However, the total number of carbon atoms shows a difference in the fragments from LANaPDB with respect to the reference libraries. The reference data set contains between 10 and 15 carbon atoms, while the LANaPDB fragments have a mean of 24. Corresponding, the mean values of the molecular-weight LANaPDB are 460.51 and the other reference databases are between 215 and 292. Though, the largest fragments are those obtained from COCONUT with an average value of molecular weight and carbon atoms, 517 and 26, respectively.

A reduction in the fraction of oxygens of reference libraries ca*.* 0.1 compared to LANaPDB was identified, finding values with a range of 0.10–0.12 for fragments and 0.8–0.9 for “Fragment RO3”. In contrast, the fraction of nitrogen increased >0.1 in reference libraries compared to the displayed value of LANaPDB, with a range of 0.12–0.16 for fragments and 0.12–0.14 for “Fragment RO3”. This is consistent with reports in the literature, the NP often has more oxygen atoms and fewer nitrogen atoms than synthetic molecules. This may be due to the fact that while nitrogen is a necessary component of nature, it is more specialized and therefore less prevalent in organic compounds, while oxygen is a frequent constituent in the biochemical reactions of NP and is so found in more metabolites.^55,57^

The carbon fractions of the 0.71 COCONUT fragments were similar to “Fragment RO3” of COCONUT 0.78. COCONUT has a similar behavior to that presented by LANaPDB in that the fraction of oxygen decreases and nitrogen increases in reference databases.

Complexity descriptors (Table 6), sp^3^, and chiral carbon fractions of COCONUT’s compounds were 0.57 and 0.18, respectively, and for LANaPDB’s compounds were 0.59 and 0.21, respectively. These values are similar between NP compounds though LANaPDB compounds were the most complex, presenting the higher mean in both descriptors. The sp^3^ and chiral carbon fractions of COCONUT’s compounds were similar to the “Fragment RO3” of COCONUT (0.55 and 0.14). A difference was observed with the COCONUT fragments with a higher fraction of sp^3^, carbons (0.64), and fraction of chiral carbons (0.29). The sp^3^ (0.6) and chiral carbon (0.17) fractions of LANaPDB compounds are more similar to the values obtained for LANaPDB’s “Fragment RO3” (0.6 and 0.17) and LANaPDB’s fragments (0.71 and 0.32).

We considered the NP data sets to be more complex than other library references. The fraction of sp^3^ and chiral carbon^42^ of fragments (sp^3^ = 0.18–0.41 and chiral = 0.01–0.04) and “Fragment RO3” (sp^3^ = 0.16–0.47 and chiral = 0.01–0.05) for NP was the highest regarding reference fragments and “Fragment RO3” of Enamine, ChemDiv, Maybridge, and Life Chemicals.

### 3.4 Structural Similarity

**Figure 6:**
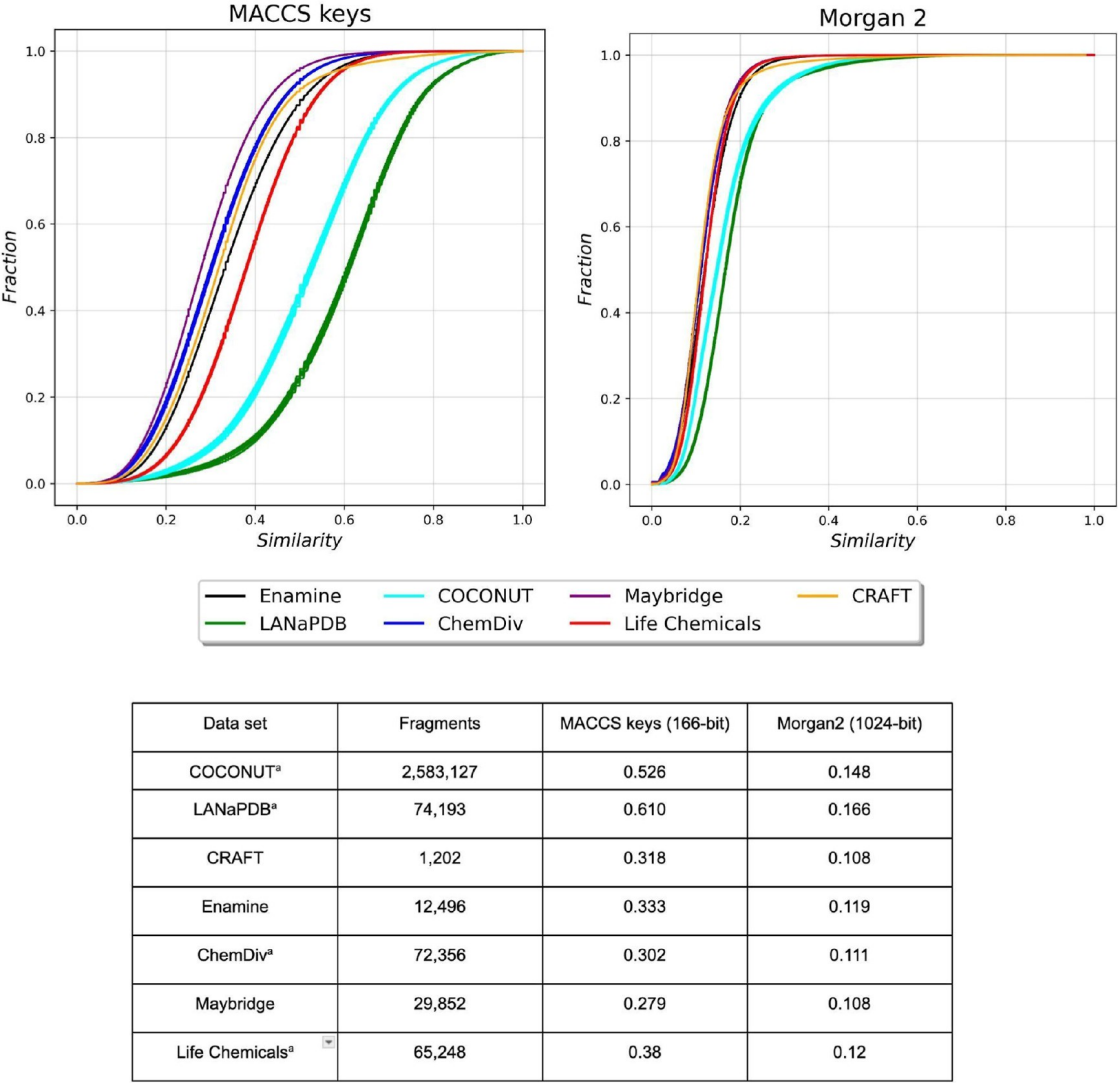
Cumulative distribution functions of the pairwise Tanimoto similarity using Morgan2 (1024 bit) and MACCS keys (166 bit) of Fragments from COCONUT (cyan), LANaPDB (green), CRAFT (yellow), Enamine (black), ChemDiv (blue), Maybridge (purple), and Life Chemicals (red). The distribution of ten subsets of 5000 fragments randomly selected from COCONUT, LANaPDB, ChemDiv, and Life Chemicals is plotted. The table summarizes the median value of the distributions.

Figure 6 shows the cumulative distribution function and summary statistics of the pairwise Tanimoto similarity using Morgan2 (1024 bit) and MACCS (166 bit) key fingerprints for the fragment libraries. The data sets are distinguished by different colors: COCONUT (cyan), LANaPDB (green), CRAFT (yellow), Enamine (black), ChemDiv (blue), Maybridge (purple), and Life Chemicals (red). Due to the large number of fragments obtained from COCONUT, LANaPDB, ChemDiv, and Life Chemicals (more than 65,248 fragments, Figure 6), ten subsets of 5000 structures were randomly selected from each data set to compute the fingerprint-based similarity. The similarity distribution of the pairwise similarity values computed with the Tanimoto coefficient and the fingerprints MACCS keys and Morgan 2, along with the summary statistics in Figure 5, indicates that Maybridge, ChemDiv, CRAFT, and Enamine were the most diverse fragment libraries, followed by Life Chemicals and the NP-based fragment libraries, COCONUT and LANaPDB. The NP-based fragments were the least diverse, which was unexpected. This could be because of the vast number of fragments and the data variability that are being studied. The distribution of the data is in a considerably wider range and the curve is pushed toward greater similarity compared to the reference libraries.

**Figure 7:**
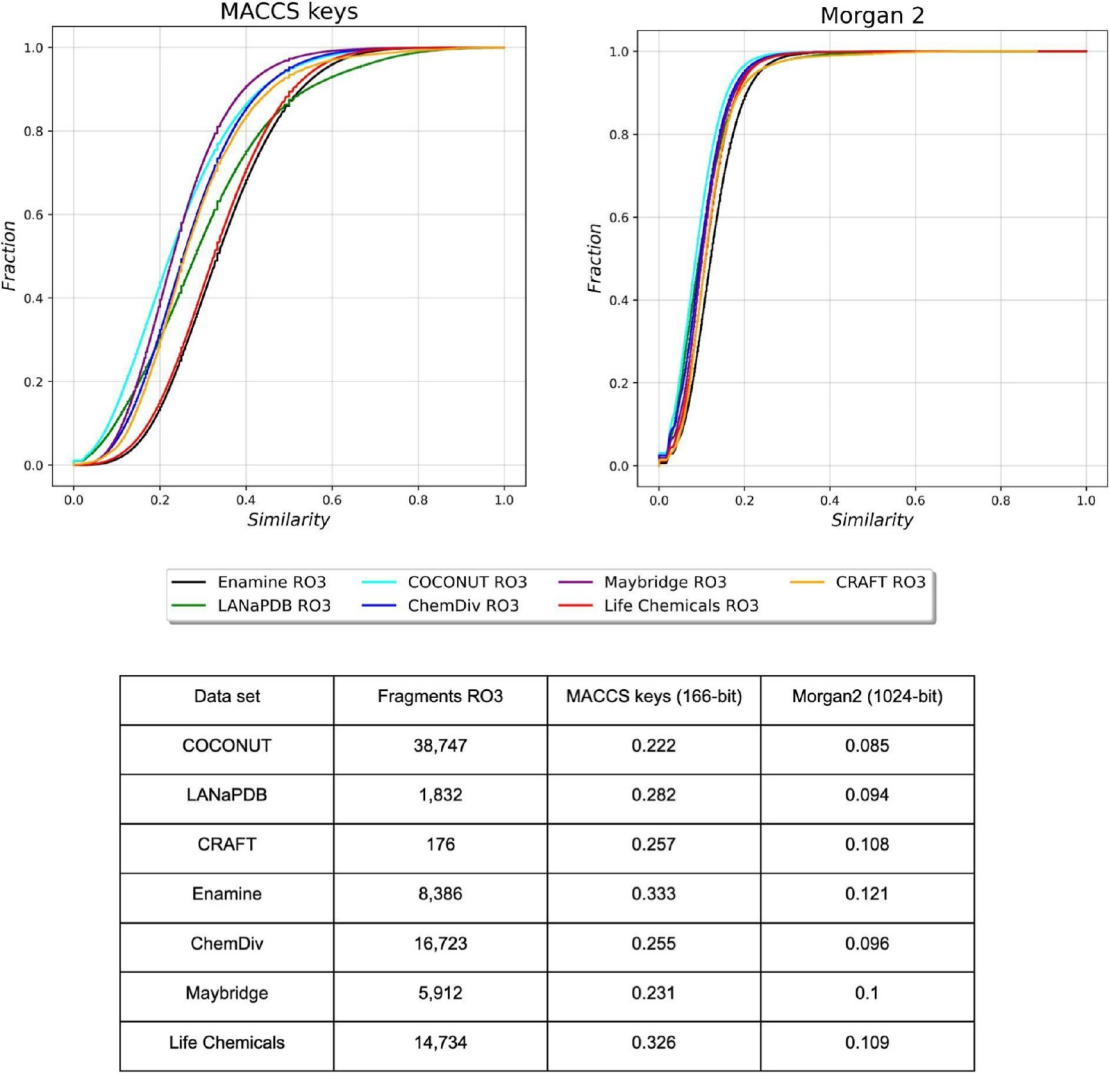
Cumulative distribution functions of the pairwise Tanimoto similarity using Morgan2 (1024 bit) and MACCS keys (166 bit) of “Fragment RO3” from COCONUT (cyan), LANaPDB (green), CRAFT (yellow), Enamine (black), ChemDiv (blue), Maybridge (purple), and Life Chemicals (red). The table summarizes the median value of the distributions.

Figure 7 shows the cumulative distribution function and summary statistics of the pairwise Tanimoto similarity using Morgan2 (1024 bit) and MACCS key (166 bit) fingerprints for the “Fragment RO3”. The data sets are color-coded using the same colors as those in Figure 6. The cumulative distribution function and summary statistics indicate that, in general, the COCONUT, Maybridge, ChemDiv, CRAFT, and LANaPDB were the most diverse “Fragment RO3” libraries, followed by Life Chemicals and Enamine. It is interesting to note that the distribution of the NPs is the most diverse and the most similar when looking at the results for the fragments and fragment RO3 (Figures 6 and 7). This suggests that focusing on the “Fragment RO3”, they capture a sizable portion of the diversity of the NPs. Notably, the structural diversity of the fragment libraries, as captured by the fingerprints, is still large and it is not compromised or restricted by the RO3 rules.

### 3.5 Synthetic Accessibility

**Figure 8:**
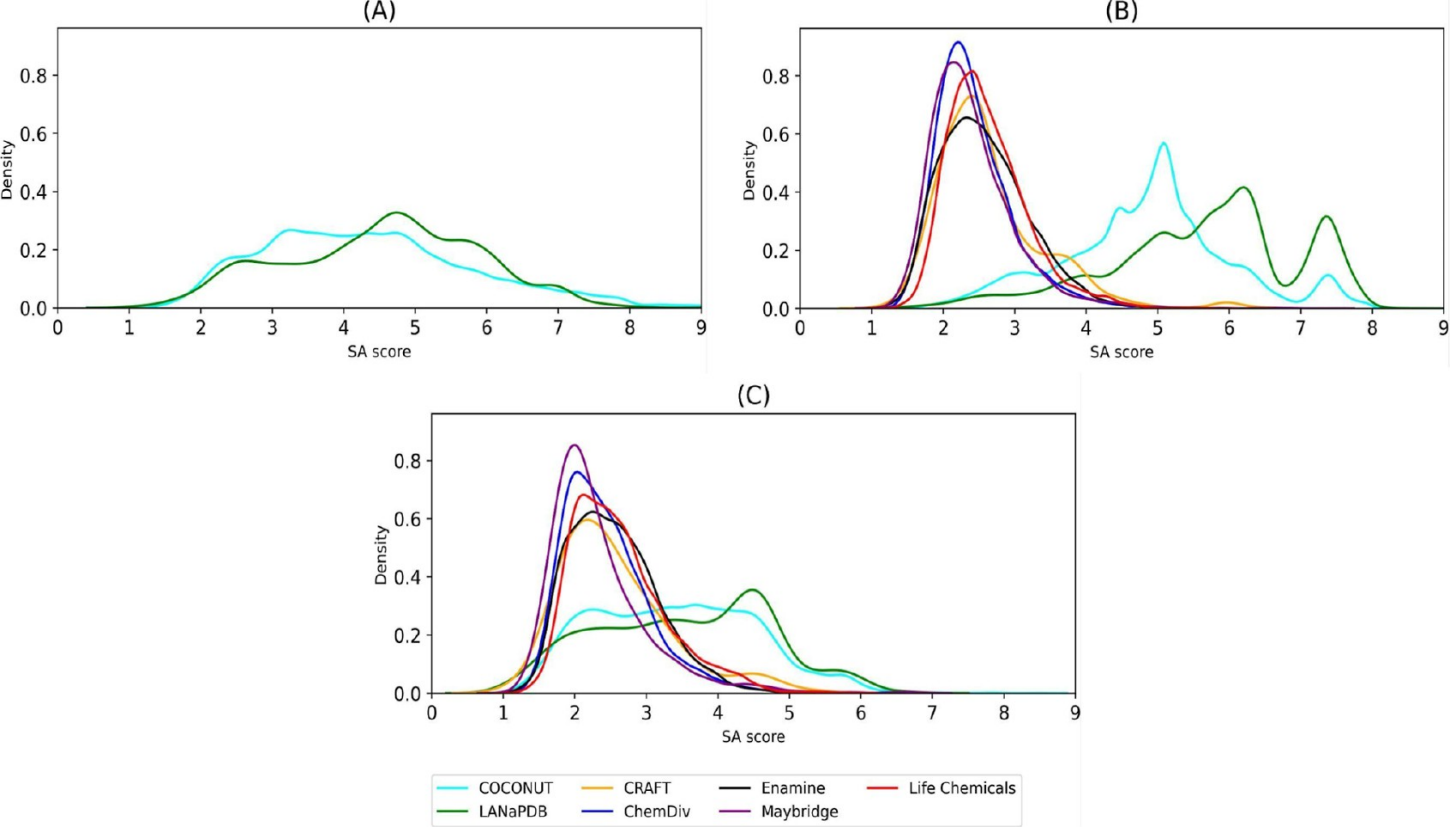
Synthetic accessibility (SA) score of (A) compounds in COCONUT (cyan) and LANaPDB (green), (B) fragment libraries, and (C) “Fragment RO3”. Data sets are shown in different colors.

Figure 8 shows the distribution of density for the SA score of compounds in LANaPDB and COCONUT (Figure 8A), fragment libraries (8B), and “Fragment RO3” (8C) for all libraries. The SA score values for compounds in NP databases are similar (for example, the mean SA score value for COCONUT is 4.85 and that for LANaPDB is 4.53). For both NP databases, 86% of the compounds are found with a SA value ≤6, meaning that this fraction of structures is easy to synthesize (according to this approximation).

The SA score values for LANaPDB and the COCONUT fragment libraries (Figure 8B) (mean values of 5.64 and 4.85, respectively) are larger than the entire compounds in the LANaPDB and the COCONUT libraries. This result could be associated with the structural complexity that is considered in the SA score, being more difficult to synthesize the entire NP due to the presence of macrocycles, fused bicycles, and bridge bicycles.^34^Figure 8B also indicates that the fragment libraries of Maybridge (mean SA score of 2.39), ChemDiv (2.43), Enamine (2.57), Life Chemicals (2.61), and CRAFT (2.65) had the lowest, most favorable SA scores. Only four molecules of the 1202 CRAFT fragments have an SA score higher than 6. It is expected that the reference fragment libraries would show more favorable SA score values, because the fragments are commercially available from various vendors. The fragments comprising the CRAFT library have been obtained experimentally based on new heterocyclic scaffolds and NP.^32^ LANaPDB and the COCONUT fragments had higher SA score values (Figure 8B). In fact, around 43% (32,039 structures) of LaNAPDB and 14% (370,800 structures) of COCONUT have SA score values larger than 6, which emphasize the structural complexity and challenge to synthesize NPs.

Overall, all “Fragment RO3” databases (Figure 8C) had a mean SA score in the range considered easy to synthesize (SA value ≤6): Maybridge (2.33), ChemDiv (2.44), Enamine (2.54), CRAFT (2.56), Life Chemicals (2.62), COCONUT (3.41), and LANaPDB (3.53). Only 1% of the “Fragment RO3” databases derived from COCONUT and LANaPDB had SA score values larger than 6. Taken together, it can be summarized that in general and as expected, “Fragments RO3” are more synthetically feasible than fragments not following RO3.

### 3.6 Visual Representation of the Chemical Space and Chemical Multiverse of Fragment RO3

**Figure 9:**
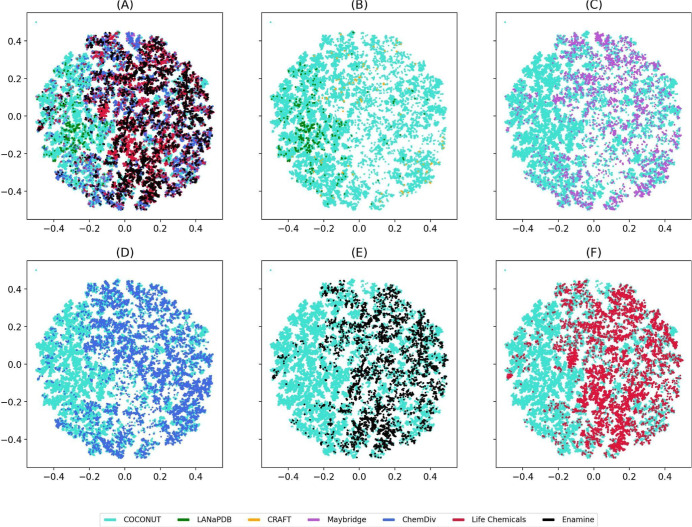
Chemical space visualization of NPs and commercial “Fragment RO3” using TMAP and MACCS keys (166 bit). Data sets are shown in different colors, as indicated in the legend. Chemical space of “Fragment RO3” was split into six panels: (A) All “Fragment RO3”; (B) COCONUT, LANaPDB, and CRAFT; (C) COCONUT and Maybridge; (D) COCONUT and ChemDiv; (E) COCONUT and Enamine; and (F) COCONUT and CRAFT.

**Figure 10:**
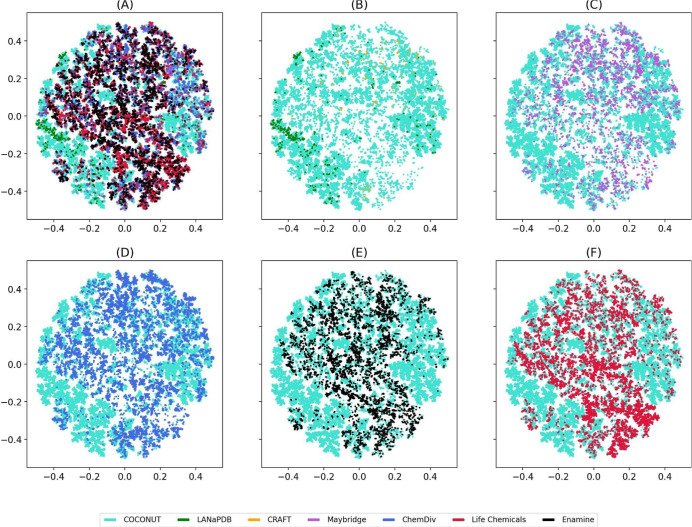
Chemical space visualization of NPs and commercial “Fragment RO3” using TMAP and Morgan2 (1024 bit). Data sets are shown in different colors, as indicated in the legend. Chemical space of “Fragment RO3” was split into six panels: (A) All “Fragment RO3”; (B) COCONUT, LANaPDB, and CRAFT; (C) COCONUT and Maybridge; (D) COCONUT and ChemDiv; (E) COCONUT and Enamine; and (F) COCONUT and CRAFT.

Figure 9 shows a visual representation of the chemical space of NPs and commercial “Fragment RO3” libraries generated with TMAP using MACCS keys. Figure 10 depicts the chemical space of the same libraries with TMAP based on Morgan2. The visualization with Morgan2 is very similar to the one generated with Morgan3 fingerprints (Figure S5 in the Supporting Information). Figures 9 and 10 libraries are represented in different colors: cyan (COCONUT), yellow (CRAFT), green (LANaPDB), black (Enamine), red (Life Chemicals), and blue (ChemDiv). The visualization of the chemical multiverse (chemical space with different representations, Figures 9 and 10) indicates that “Fragments RO3” from COCONUT cover a large region of the space followed by ChemDiv, Life Chemicals, Enamine, LANaPDB, and CRAFT. This is in line with the quantitative diversity analysis that shows that CRAFT was the fourth most diverse “Fragment RO3” library according to MACCS keys and Morgan2 (Figure 7).

Figures S6, S7, and S8 show a visual representation of the chemical space of the “Fragment RO3” libraries using t-SNE, MACCS keys, and Morgan2 and Morgan3 (Figures S6, S7, and S8 in the Supporting Information). The visualization with Morgan2 (Figure S7) is very similar to the one generated with Morgan3 fingerprints (Figure S8). In agreement with the TMAPs (Figure 8), “Fragments RO3” of COCONUT cover a large surface of the chemical space generated with MACCS keys (Figure S6), Morgan2 (Figure S7), and Morgan3 (Figure S8). Figures 9B–F and 10B–F indicate that “Fragments RO3” of LANaPDB, CRAFT, Maybridge, ChemDiv, Enamine, and Life Chemicals share the same chemical space as “Fragments RO3” of COCONUT. However, “Fragments RO3” of LANaPDB and CRAFT share chemical space regions different from those of “Fragments RO3” of Maybridge, ChemDiv, Enamine, and Life Chemicals. These results mean that although the CRAFT fragments are synthetic and NP inspired,^32^ they retain similar structural features from NP fragments and are to be expected to overlap with the chemical space of NP fragments like COCONUT and LANaPDB. In general, t-SNE and TMAP had similar results. Still, the advantage of TMAP^48^ in contrast with t-SNE is preserving all information about the chemical structures of fragments as possible. t-SNE is a nonlinear dimension reduction method,^49^ and more information is lost based on the number of descriptors used.

These results underscore the significant structural diversity offered by natural products, suggesting their potential as a rich source for the discovery of novel compounds in drug development. NPs have historically played a crucial role in the development of many therapeutic agents, accounting for a substantial percentage of drugs currently in use.^58^ This diversity not only provides a vast array of unique scaffolds but also facilitates the identification of compounds with distinct biological activities.^59^ Furthermore, recent advances in techniques such as high-throughput screening and cheminformatics have enhanced our ability to explore the vast chemical space represented by natural products, leading to the identification of promising candidates for various therapeutic targets and different neglected and emerging diseases.^60−63^

## 4 Conclusions

Herein, we analyzed the contents, properties, and chemical diversity of fragment libraries obtained from the latest releases of the COCONUT and LANaPDB, CRAFT, and commercial fragment libraries. It was concluded that NPs have the highest percentage of unique fragments and “Fragment RO3”. These results highlight the great structural diversity provided by the NPs. Also, NP fragments had higher values of sp^3^ and fraction of chiral carbons than the reference fragment libraries ChemDiv, Maybridge, Enamine, and Life Chemicals. Similarly, CRAFT fragments and “Fragment RO3” had the highest percentage of unique fragments as compared to the fragment libraries derived from COCONUT and LANaPDB. It was also found that the fragments from NPs (including the one that complies with the RO3) had a similar number of heterocycles and carbons and similar values of nitrogens, oxygens, sp^3^, and chiral carbon fraction, meaning that NP fragments retained structural features and complexity features of NPs from which they are derived. We also found that “Fragments RO3” from COCONUT were the most structurally diverse (as quantified using molecular fingerprints), meaning that the filters of the RO3 reduced the total number of COCONUT fragments but the COCONUT fragments that comply with the RO3 have a high diversity. All fragment libraries herein obtained and curated are freely available at https://github.com/DIFACQUIM/Fragment-libraries-from-large-synthetic-compounds-and-natural-products-collections.git.

## Supporting Information Available

Unique and common fragments between CRAFT, LANaPDB, COCONUT, and reference libraries; cumulative distribution functions of the pairwise Tanimoto similarity using Morgan3 of Fragments and “Fragments RO3” from COCONUT, LANaPDB, CRAFT, Enamine, ChemDiv, Maybridge, and Life Chemicals; and chemical space visualization of commercial “Fragments RO3” using TMAP and t-SNE with Morgan3 and MACCS keys (PDF)

## Abbreviations

BRICS: Breaking of Retrosynthetically Interesting Chemical Substructures
CRAFT: Center for Research and Advancements in Fragments and Molecular Targets
COCONUT: Collection of Open Natural Products
FBDD: fragment-based drug discovery
HBAs: hydrogen-bond acceptors
HBDs: hydrogen-bond donors
LANaPDB: Latin America Natural Product Database
Log P: partition coefficient octanol/water
NPs: natural products
MACCS: Molecular ACCes System
MORTAR: MOlecule fRagmenTAtion fRamework
MW: molecular weight
RECAP: Retrosynthetic Combinatorial Analysis Procedure
RB: rotatable bonds
SA: synthetic accessibility
SMILES: Simplified Molecular Input Line Entry System
TMAP: Tree MAP
TPSA: topological polar surface area.
