# Supplementary material for: Fragment Libraries from Large and Novel Synthetic Compounds and Natural Products: A Comparative Chemoinformatic Analysis
Source: ACS Omega. 2025 Apr 16;10(16):16921–37. doi: 10.1021/acsomega.5c01420 (PMC12044453; doi:10.1021/acsomega.5c01420)
Supplement: Supplementary file 1 — ao5c01420_si_001.pdf [file ao5c01420_si_001.pdf]

SUPPLEMENTARY MATERIAL

Fragment libraries from large and novel synthetic compounds and natural products: A comparative chemoinformatic analysis

Verónica Ramírez-Cid[1], Ana L. Chávez-Hernández[1], Osvaldo Sánchez López[1], Raul Marques Novais[2], Temitayo Omowumi Alegbejo Price[2], Kamilla Moraes Alves[2,3], Wemenes J. Lima Silva[2,3], Flavio da Silva Emery[2], Carolina Horta Andrade[2,3,4*], José L. Medina-Franco[1*]

[1]DIFACQUIM Research Group, Department of Pharmacy, School of Chemistry, Universidad Nacional Autónoma de México, Mexico City 04510, Mexico

[2]Center for Research and Advancement in Fragments and Molecular Targets (CRAFT), School of Pharmaceutical Sciences at Ribeirao Preto, University of São Paulo, Ribeirão Preto, São Paulo 05508-060, Brazil.

[3]Laboratory for Molecular Modeling and Drug Design (LabMol), Faculty of Pharmacy, Universidade Federal de Goiás, Goiânia, Goiás 74605-170, Brazil.

[4]Center for Excellence in Artificial Intelligence (CEIA), Institute of Informatics, Universidade Federal de Goiás, Goiânia, Goiás 74605-170, Brazil.

* Contact authors: medinajl@unam.mx; Tel: +52 (55) 5622-3899, ext. 44458 (JLMF); craft@usp.br; carolina@ufg.br; Tel: +55 62 3209-6451 (CHA).

Contents

		Page
**Figure S1**	Unique and common fragments between CRAFT (yellow) and reference library (purple).	S2
**Figure S2**	Unique and common fragments between LANaPDB (yellow) and reference library (purple).	S3
**Figure S3**	Unique and common fragments between COCONUT (yellow) and reference library (purple).	S4
**Figure S4**	Cumulative distribution functions of the pairwise Tanimoto similarity using Morgan3 (1024-bit) of Fragments and “Fragments RO3” from COCONUT, LANaPDB, CRAFT, Enamine, ChemDiv, Maybridge and Life Chemicals.	S5
**Figure S5**	Chemical space visualization of commercial “Fragments RO3” using TMAP and Morgan3 (1024-bit).	S6
**Figure S6**	Chemical space visualization of commercial “Fragments RO3” using t-SNE and MACCS keys (166-bit).	S7
**Figure S7**	Chemical space visualization of commercial “Fragments RO3” using t-SNE and Morgan2 (1024-bit).	S8
**Figure S8**	Chemical space visualization of commercial “Fragments RO3” using t-SNE and Morgan3 (1024-bit).	S9

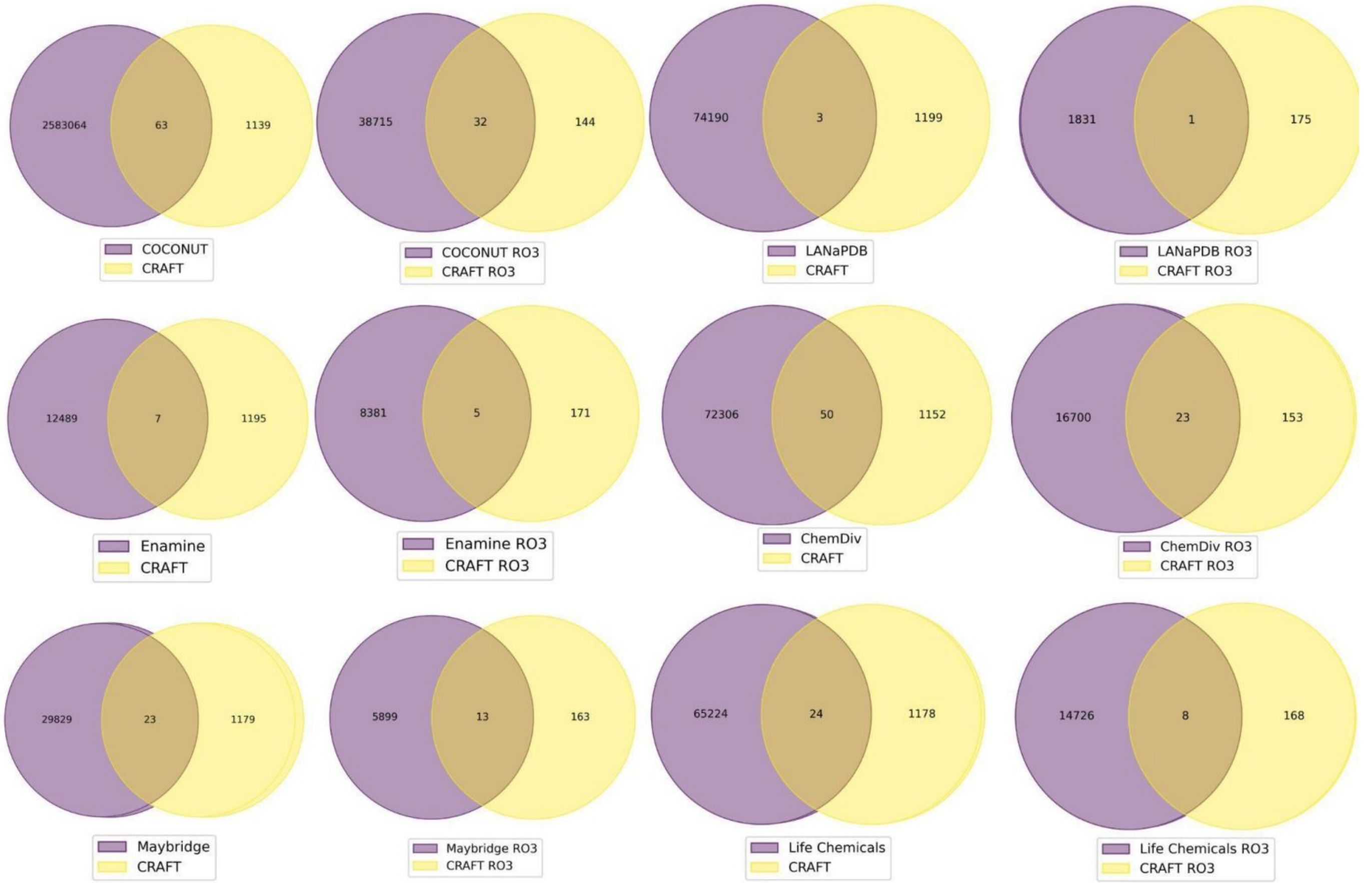

Figure S1. Unique and overlapping fragments between CRAFT (yellow) and reference library (purple).

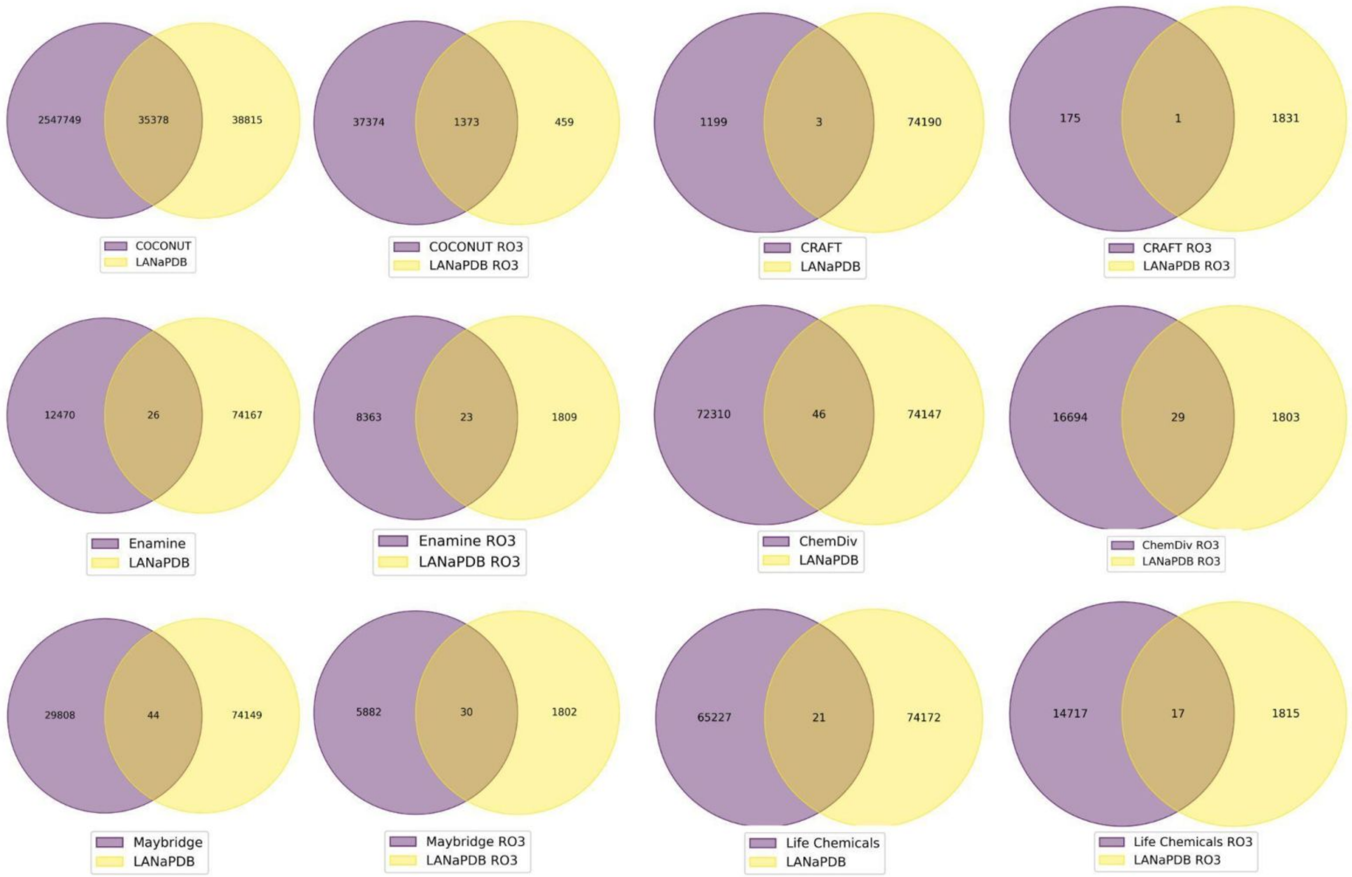

Figure S2. Unique and overlapping fragments between LANaPDB (yellow) and reference library (purple).

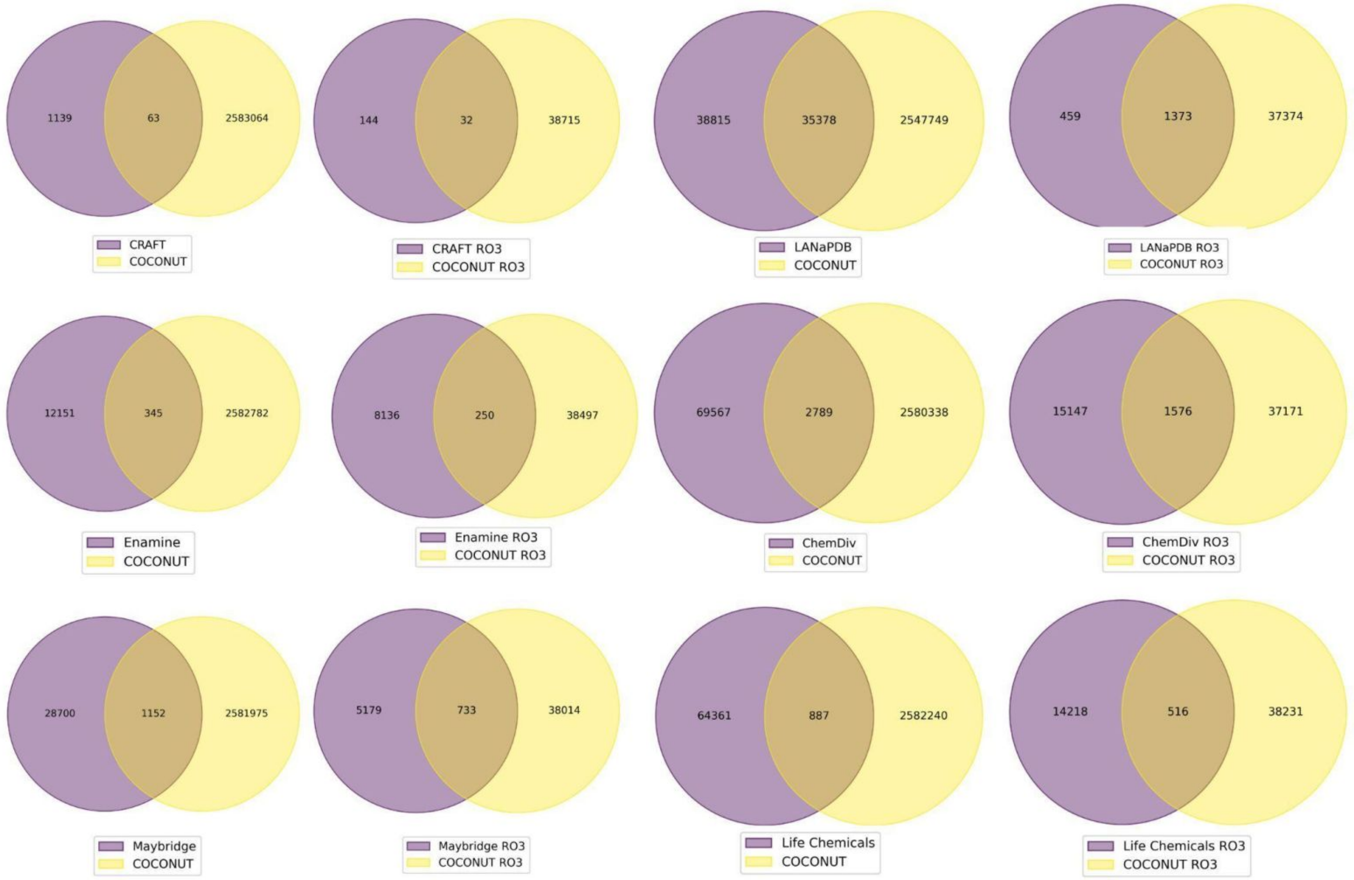

Figure S3. Unique and overlapping fragments between COCONUT (yellow) and reference library (purple).

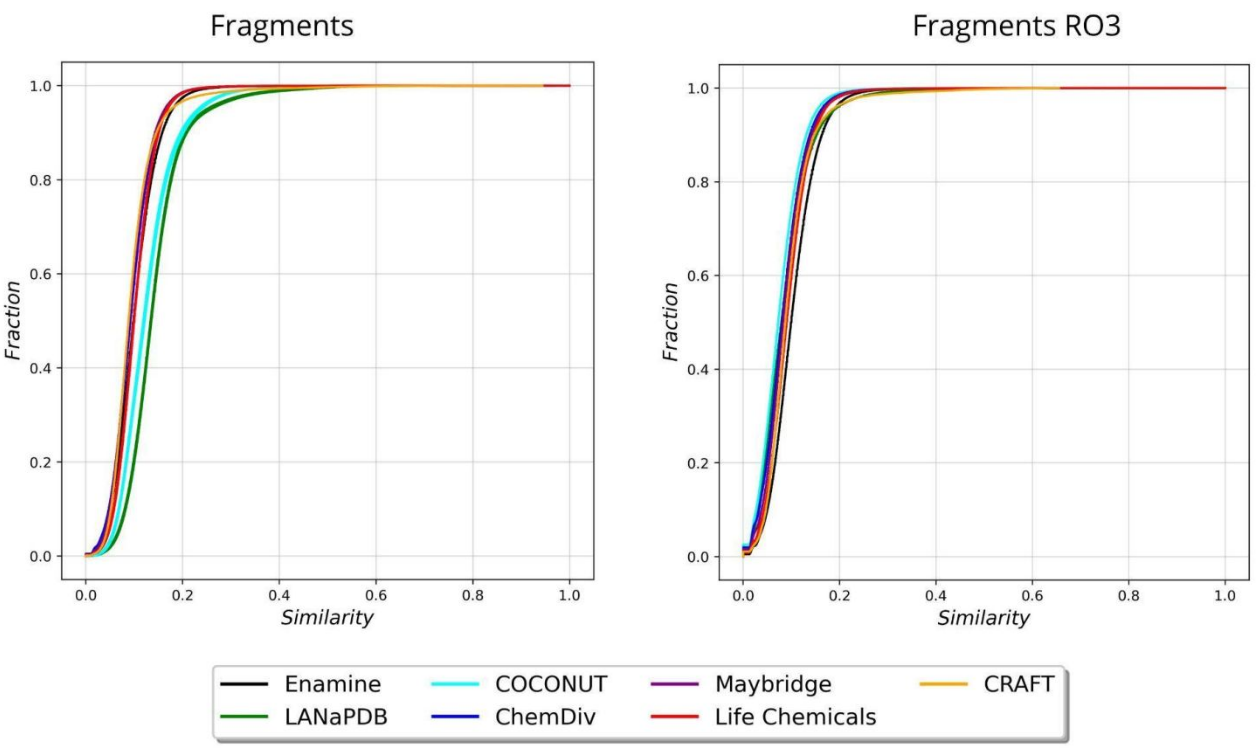

Data set	Fragments	Fragments RO3	Morgan3 (1024-bit) Fragments	Morgan3 (1024-bit) Fragments RO3
COCONUT[a]	2,583,127	38,747	0.12	0.072
LANaPDB[a]	74,193	1,832	0.135	0.079
CRAFT	1,202	176	0.088	0.087
Enamine	12,496	8,386	0.098	0.099
ChemDiv[a]	72,356	16,723	0.092	0.08
Maybridge	29,852	5,912	0.089	0.082
Life Chemicals[a]	65,248	14,734	0.099	0.09

Figure S4. Cumulative distribution functions of the pairwise Tanimoto similarity using Morgan3 (1024-bit) of Fragments and “Fragments RO3” from COCONUT (cyan), LANaPDB (green), CRAFT (yellow), Enamine (black), ChemDiv (blue), Maybridge (purple) and Life Chemicals (red). This table summarizes the median value of the distributions.

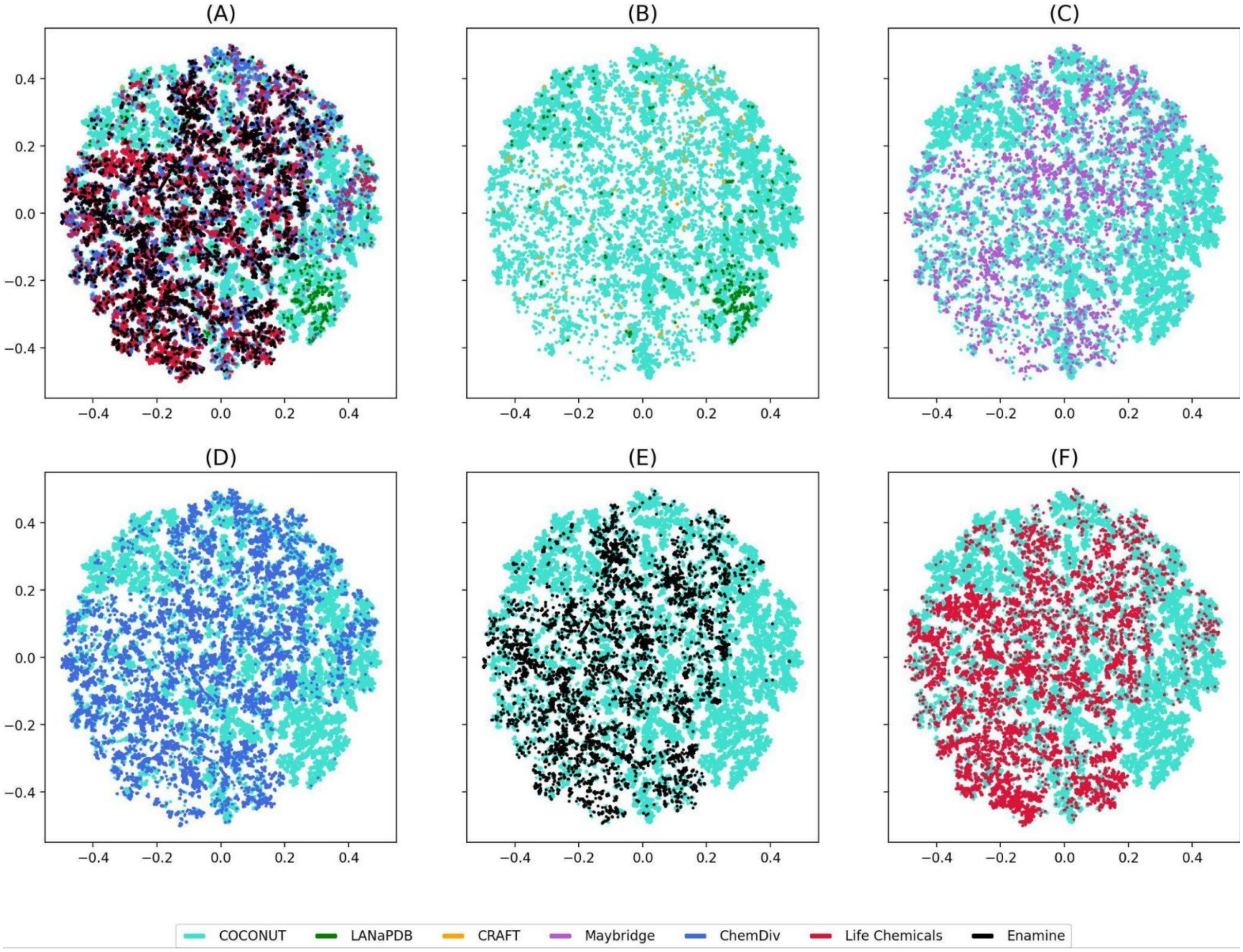

Figure S5. Chemical space visualization of NP and commercial "Fragments RO3" using TMAP and Morgan3 (1024-bit). Datasets are shown in different colors, as indicated in the legend. Chemical space of "Fragments RO3" was split into six panels: (A) All "Fragments RO3"; (B) COCONUT, LANaPDB and CRAFT; (C) COCONUT and Maybridge; (D) COCONUT and ChemDiv; (E) COCONUT and Enamine; (F) COCONUT and CRAFT.

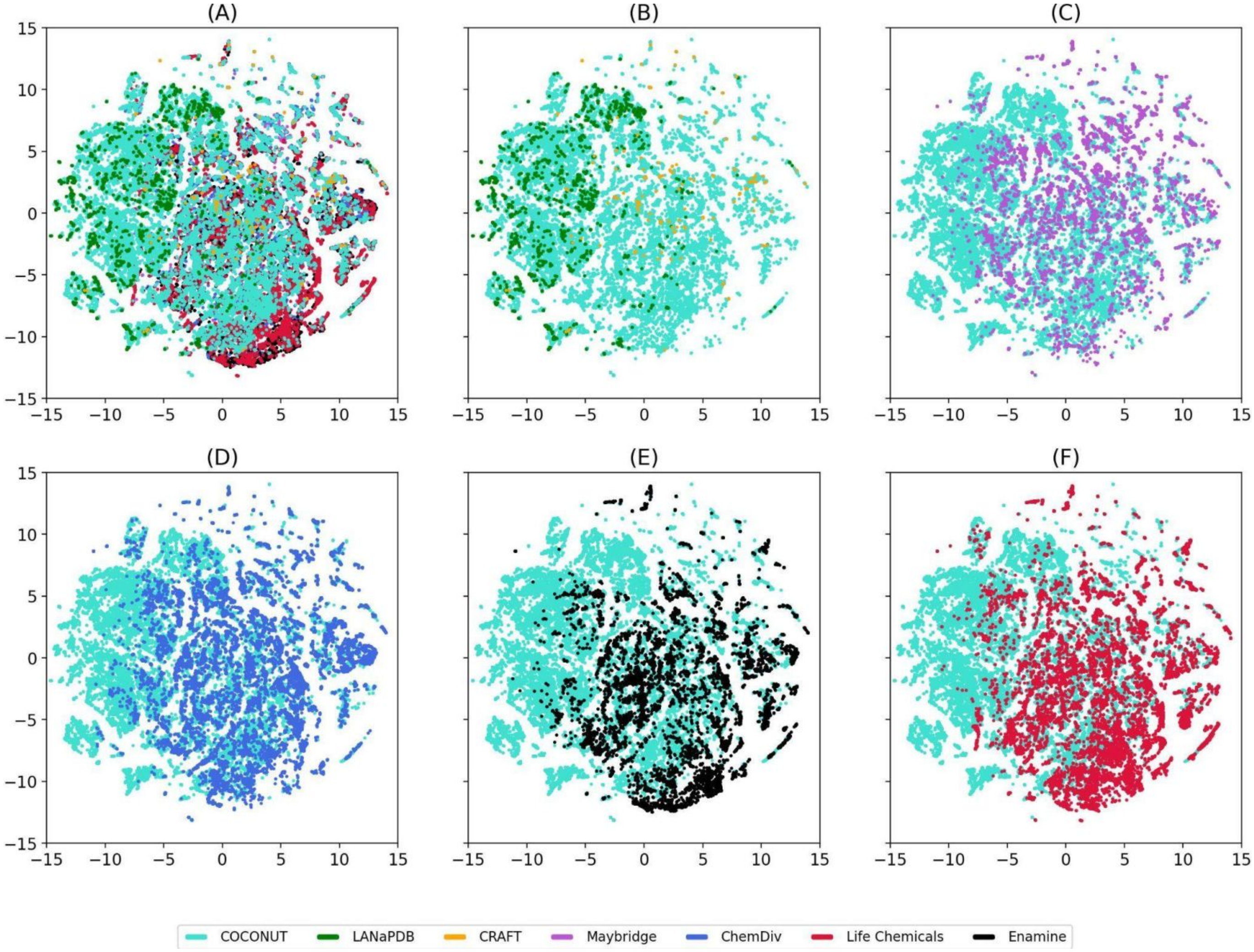

Figure S6. Chemical space visualization of NP and commercial “Fragments RO3” using t-SNE and MACCS keys (166-bit). Datasets are shown in different colors, as indicated in the legend. Chemical space of “Fragments RO3” was split into six panels: (A) All “Fragments RO3”; (B) COCONUT, LANaPDB and CRAFT; (C) COCONUT and Maybridge; (D) COCONUT and ChemDiv; (E) COCONUT and Enamine; (F) COCONUT and CRAFT.

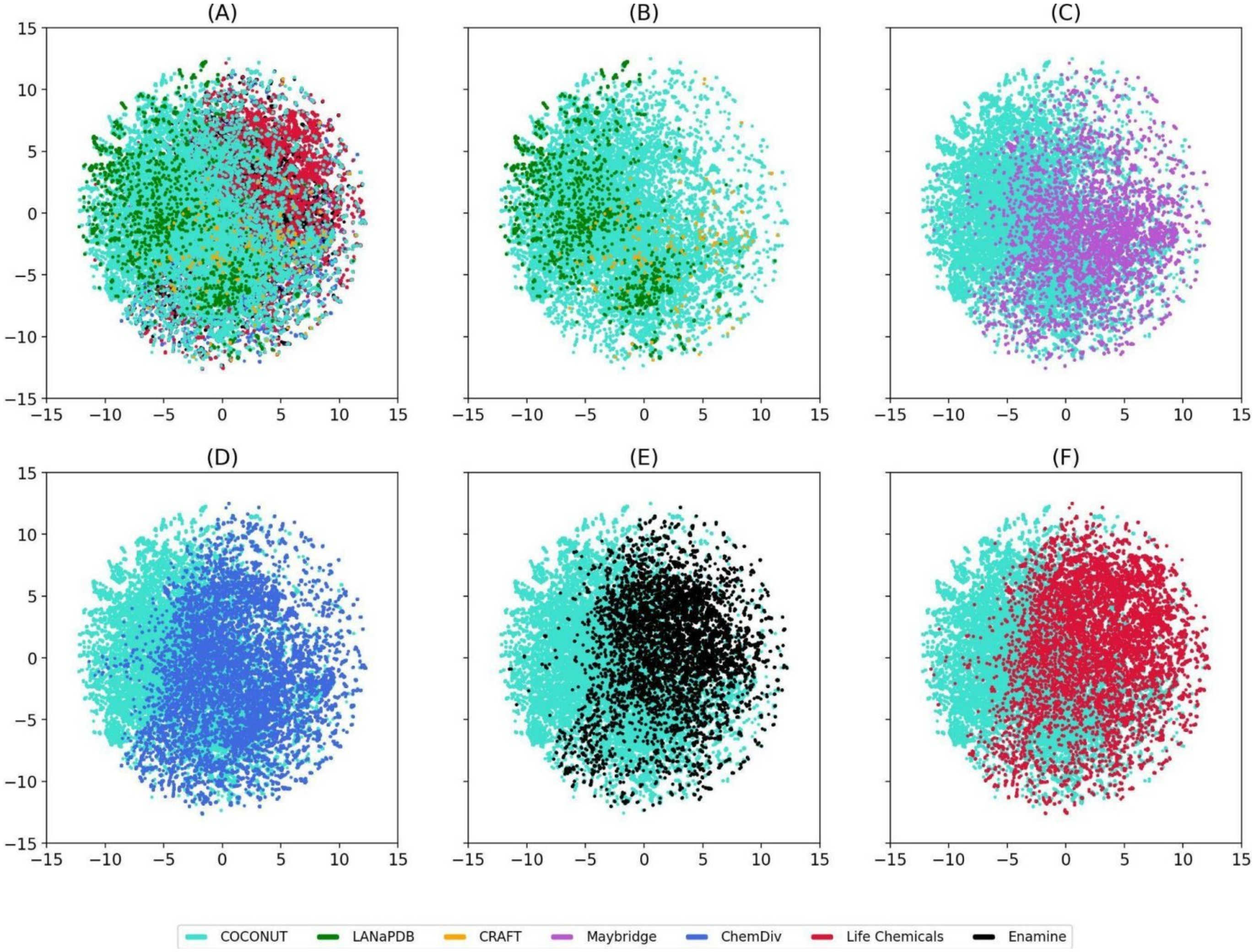

Figure S7. Chemical space visualization of commercial “Fragments RO3” using t-SNE and Morgan2 (1024-bit). Datasets are shown in different colors, as indicated in the legend. Chemical space of “Fragments RO3” was split into six panels: (A) All “Fragments RO3”; (B) COCONUT, LANaPDB and CRAFT; (C) COCONUT and Maybridge; (D) COCONUT and ChemDiv; (E) COCONUT and Enamine; (F) COCONUT and CRAFT.

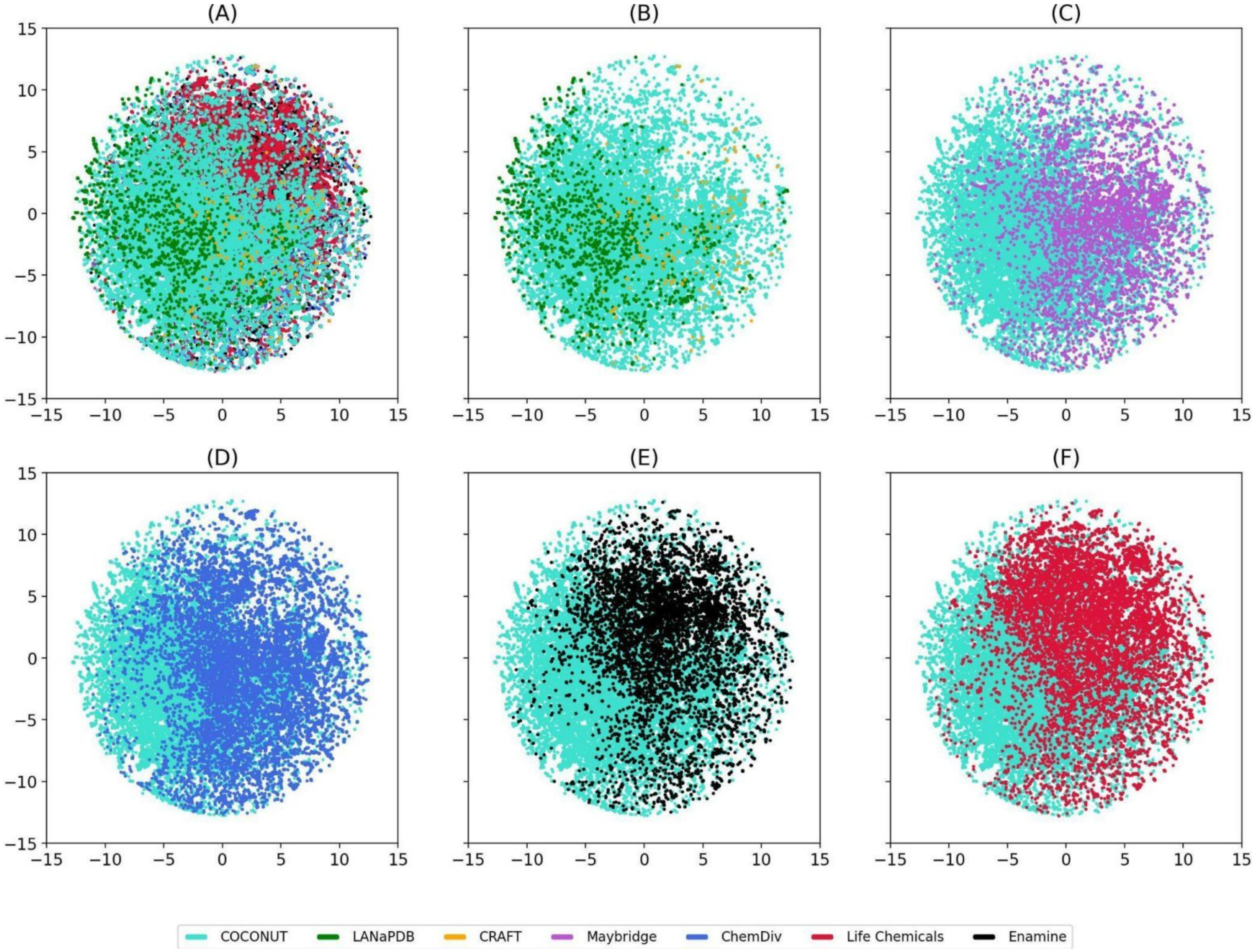

Figure S8. Chemical space visualization of commercial "Fragments RO3" using t-SNE and Morgan3 (1024-bit). Datasets are shown in different colors, as indicated in the legend. Chemical space of "Fragments RO3" was split into six panels: (A) All "Fragments RO3"; (B) COCONUT, LANaPDB and CRAFT; (C) COCONUT and Maybridge; (D) COCONUT and ChemDiv; (E) COCONUT and Enamine; (F) COCONUT and CRAFT.
